# Trismus among Preclinical Students in a Medical College: A Descriptive Cross-sectional Study

**DOI:** 10.31729/jnma.7900

**Published:** 2022-11-30

**Authors:** Nripendra Tiwari

**Affiliations:** 1 Department of Anatomy, Kathmandu Medical College and Teaching Hospital, Sinamangal, Kathmandu, Nepal

**Keywords:** *prevalence*, *temporomandibular joint*, *trismus*

## Abstract

**Introduction:**

Trismus is a condition of reduced mouth opening due to tonic constrictions of the muscles of mastication. Trismus greatly affects health-related quality of life and thus daily life activities. The aim of this study was to find out the prevalence of trismus among preclinical students in a medical college at a tertiary care teaching hospital.

**Methods:**

A descriptive cross-sectional study was conducted in a medical college from 10 July 2021 to 10 December 2021 for a period of five months. Ethical clearance was obtained from the Institutional Research Committee (Reference number: 0311202004). A total of 315 preclinical students studying at a medical college were included in the current study. Convenience sampling method was used. Point estimate and 99% Confidence Interval were calculated.

**Results:**

Among 315 students, trismus was seen in 14 (4.44%) (1.45-7.43, 95% Confidence Interval). The clicking sound on the right side of the temporomandibular joint was found to be in 6 (42.86%), on the left side was 5 (35.71%) and that of both sides of the temporomandibular joint was 1 (7.14%) among the 14 students with trismus. The mean maximal interincisal mouth opening was found to be 33.4±0.46 mm in students having trismus.

**Conclusions:**

The prevalence of trismus was found to be similar to the other studies performed in similar settings among preclinical students in a medical college. Awareness on mouth opening exercises and timely management can help reduce trismus.

## INTRODUCTION

Trismus is a condition where there is limited mouth opening due to tonic constractions of the muscles of mastication. It has got an important impact on health-related quality of life with temporomandibular jaw-related problems, eating limitations, muscular pain and tension.^1^ The maximum interincisal opening in reduced mandible mobility or trismus is less than or equal to 35 mm on average in adults according to Gothenburg Trismus Questionnaire (GTQ).^2^

Persons with poor oral hygiene, jaw-related problems, diet limitations, excessive chewing habits, muscular tension, disorders of tonsils and immediately having a difficult level of tooth extraction are more prone to develop trismus.^3^ Preoperatively verification of the level of trismus would prevent further injuries and complications.^4^ Particular attention preoperatively should be given to the mouth opening limit of the individual to avoid further complications.^5^

The aim of this study was to find the prevalence of trismus among preclinical students in a medical college.

## METHODS

A descriptive cross-sectional study was conducted in the Department of Anatomy from 10 July 2021 to 10 December 2021 for a period of five months. Ethical clearance was obtained from the Institutional Research Committee (Reference number: 0311202004). The study included all the preclinical students of Bachelor of Medicine Bachelor of Surgery (MBBS) I, MBBS II, Bachelor of Dental Surgery (BDS) I, BDS II, Bachelor of Nursing (B.Sc Nursing) and Bachelor of Nursing Science (BNS) streams, who gave consent for their participation in the research irrespective of age, sex and race at Kathmandu Medical College Teaching Hospital. The students who did not give consent for their participation in the research work were excluded from the study. Convenience sampling method was used. The sample size calculated according to the following formula:


$$\begin{array}{ll} \text{n} & ={\text{Z}}^{2}\times \frac{\text{p}\times \text{q}}{{\text{e}}^{\text{2}}}\\ & =2.576^{2}\times \frac{0.50\times 0.50}{0.10^{2}}\\ & =166 \end{array}$$

Where,

n= minimum required sample size
Z= 2.576 at 99% Confidence Interval (CI)
p= past prevalence of reduced mandibular mobility or trismus taken as 50%
q= 1-p
e= margin of error, 10%

The calculated sample size was 166. However, an examination was performed on 315 preclinical students to observe and record the trismus. Maximal Interincisal mouth opening in reference to midline between upper and lower central incisors with a value of 35 mm or less than 35 mm was labelled as the reduced mandibular mobility or trismus condition according to Gothenburg Trismus Questionnaire (GTQ). Instruments for examination used were a mouth mirror, probe, metallic divider, calibrated scale and 0.01 mm sensitive digital caliper to measure various dimensions. Both sides of the temporomandibular joints of the subjects were palpated over a preauricular area, anterior to the tragus of the ear after taking written consent to observe clicking sounds or the jaw noises on right, left and both sides of Temporomandibular Joint (TMJ) among preclinical students of MBBS I, MBBS II, BDS I, BDS II, B.Sc Nursing and BNS streams studying at the medical college. The students were asked to slowly open their mouths as wide as possible three times. The dentition was in maximum intercuspation after each closure of the mouth. Clicking or TMJ sounds on opening or closing of the mouth as per detection by palpation were recorded. The maximal interincisal opening was measured with a digital calliper, metallic divider and metallic scale between the edges of the incisors of the maxilla and the mandible. Sterilised mouth mirrors, probes, and metallic instruments were used to control cross-infection among subjects. The research was conducted in the free time of a group of 10 students without hampering the teaching and learning activities at the institute. Data was collected by a self-designed questionnaire in written form to obtain the necessary information on the variables of the study.

Data collected was compiled in Microsoft Office Excel 2013 and further analyzed by IBM SPSS Statistics version 20. Point estimate and 99% CI were calculated.

## RESULTS

Out of 315 preclinical students, the prevalence of trismus was found to be 14 (4.44%) (1.45-7.43, 95% CI). The clicking sound on the right side TMJ was found to be 6 (42.86%), on the left side was 5 (35.71%) and that of both sides of TMJ was 1 (7.14%) among the 14 students with trismus (Table 1).

**Table 1:** Distribution of Clicking sound or Jaw noises, while opening and closing the mouth among preclinical students having trismus (n= 14).

Parameters	n (%)
Right side temporomandibular joint	6 (42.86)
Left side temporomandibular joint	5 (35.71)
Both sides temporomandibular joint	1 (7.14)
None	2 (14.29)

The mean maximal interincisal opening was found to be 33.4±0.46 mm among 14 students having trismus. The maximum interincisal mouth opening was found to be 33.98 mm and that of the minimum interincisal mouth opening was 32.51±1.25 mm in subjects with trismus (Table 2).

**Table 2:** Mean maximal mouth opening performed by subjects with trismus in the study population (n= 14).

Parameters	Value (mm)
Mean Maximal interincisal opening	33.4±0.46
Maximum interincisal opening	33.98
Minimum interincisal opening	32.51

## DISCUSSION

This study showed that the trismus was prevalent in 14 (4.44%) among 315 preclinical students at the medical college. A similar result was found with a prevalence of 9% in a study conducted at Sahlgrenska University Hospital, Gothenburg.^6^ A higher prevalence of trismus of 23.6% was found in research conducted on patients with head and neck cancer among 730 patients.^7^ As per the research carried on Sweden,^8^ the result for the prevalence of trismus was in contrast with the current study. They found the result of trismus as high as 42% in head and neck cancer patients and 25% in patients undergoing radiotherapy. A systemic review of trismus induced by cancer therapies in head and neck cancer patients^9^ showed a prevalence of 5% for the few-intensity modulated radiation therapy and a contrast of 25.4% in patients who received controlled radiotherapy. The cause for the difference in these results could be due to differences in the sample size, age, sex of the study population and research being done on the diseased condition of individuals suffering from head and neck cancer, undergoing chemotherapy and radiotherapy. The clicking sound or jaw noises on the right side of TMJ was found to be in 6 (42.86%), on the left side was 5 (35.71%) and that of both sides of TMJ was 1 (7.14%) among the 14 students with suffering from trismus. These results are inconsistent with the research carried out on the Italian population sample,^10^ of who found the clicking sound of TMJ on 30.7% of the Italian population. However, the clicking sound of TMJ on the right side left side and both sides were not differentiated in that research. The contrast result of some kind of joint sound was recorded in 56% as per research conducted on individuals in Sweden.^11^ The mean maximal interincisal opening in subjects with having trismus was found to be 33.4±0.46 mm.

Similar results with a slight difference were also found in research conducted with mean incisal opening improvement based on an exercise in the intervention group and comparing it with a control group.^12^

The limitation of the current study was that the cause for reduced mandibular mobility with its other associated risk factors and adverse habits like smoking, and betel nut chewing was not traced with gender differences of trismus and maximal interincisal opening. Better instruments and technologies need to be incorporated in future studies to differentiate the clicking sound of TMJ either because of disc displacement or muscular incoordination.

## CONCLUSIONS

The prevalence of trismus was found to be similar to the other studies performed in similar settings among preclinical students in a medical college. Awareness of mouth-opening exercises, physiotherapy and timely management can help reduce trismus.
